# A uniquely human sebum lipid drives streptococcal skin infection severity

**DOI:** 10.1128/mbio.01856-26

**Published:** 2026-09-03

**Authors:** Doris L. LaRock, Christopher N. LaRock

**Affiliations:** 1 Department of Microbiology and Immunology, Emory University School of Medicine 12239 , Atlanta, Georgia, USA; 2 Department of Medicine Division of Infectious Disease, Emory University School of Medicine 12239 , Atlanta, Georgia, USA; NYU Langone Health, New York, New York, USA

**Keywords:** group A *Streptococcus*, *Streptococcus pyogenes*, streptolysin, fatty acids, pathogenesis

## Abstract

Free fatty acids (FAs) secreted by the sebaceous glands are critical components of the skin barrier. Here, we investigate the activity of the major FA species present in the skin against *Streptococcus pyogenes* (group A *Streptococcus* [GAS]), a major cause of skin infections, such as impetigo, erysipelas, cellulitis, and necrotizing fasciitis. Several relevant FAs had antimicrobial activity against GAS, and linoleic acid was found to be therapeutic when applied in a murine skin infection model. However, sapienic acid, a FA abundant only in human sebum, significantly worsened disease severity despite possessing antimicrobial activity. Transcriptional profiling and molecular genetic analysis showed that sapienic acid, but not other structurally similar FAs, induced the *sag* operon. The *sag* operon encodes streptolysin S (SLS), and sapienic acid induction of this cytolytic toxin significantly increased hemolysis of human red blood cells by GAS. Screening of additional FAs identified forms with combined antimicrobial and anti-lytic activity useful as therapeutics. Taken together, we report a species- and tissue-specific trigger for GAS virulence and limitations to the use of FAs as therapeutics against infectious disease.

**IMPORTANCE:**

GAS naturally only infects humans. Here, we report that the human-specific sebum lipid sapienic acid induces production of streptolysin S (SLS), the hemolysin responsible for the hallmark β-hemolytic phenotype of GAS. Reliance on detection of a human-specific FA for expression of a critical virulence factor exposes a vulnerability of GAS and suggests a potentially variable role for SLS at different infection sites. Furthermore, it details a limitation of existing infection models, which all lack sapienic acid, for understanding the role of SLS in disease.

## OBSERVATION

Keratinocyte- and sebocyte-secreted free fatty acids (FAs) form a lipid matrix for the stratum corneum that provides impermeability to the skin and protects against invasion by pathogenic bacteria (1). For example, mice mutant for stearoyl-coenzyme A desaturase 1 (Scd1) do not produce oleic and palmitoleic acids in sebum and are susceptible to skin infection (2). FAs can be antimicrobial (3–7); *Streptococcus pyogenes* (group A *Streptococcus* [GAS]) is known to be killed by linoleic acid and, to a lesser extent, by oleic acid (2, 8, 9). Since colonization and infection of the skin are important in the lifestyle of GAS (10), we reasoned that endogenous FA may affect GAS pathogenesis and that their antimicrobial properties could make FA supplementation useful in the treatment of disease.

Sapienic and palmitic acids predominate among skin FA, followed by decreasing abundance of oleic, myristic, stearic, and linoleic acids (1, 5, 11). By minimal inhibitory concentration (MIC) assay, GAS was resistant to killing by cholesterol, another major lipid of the skin, as well as most of the most abundant species, including oleic, palmitic, myristic, lauric, and stearic acids (Fig. 1A). However, GAS was efficiently killed by linoleic acid and sapienic acid, the latter of which is specific to humans and the major FA comprising sebum (Fig. 1A). Application of these antimicrobial lipids during a GAS epicutaneous infection of mice led to reductions of bacterial burden by linoleic acid and not sapienic acid within 48 h (Fig. S1A), and pronounced skin reddening suggestive of increased inflammation during sapienic acid treatment (Fig. S1B). Since sapienic acid itself is not inflammatory toward mice (12), we hypothesized that inflammation disproportionate to bacterial burden could be due to sapienic acid inducing GAS toxins or virulence factors.

**Fig 1:**
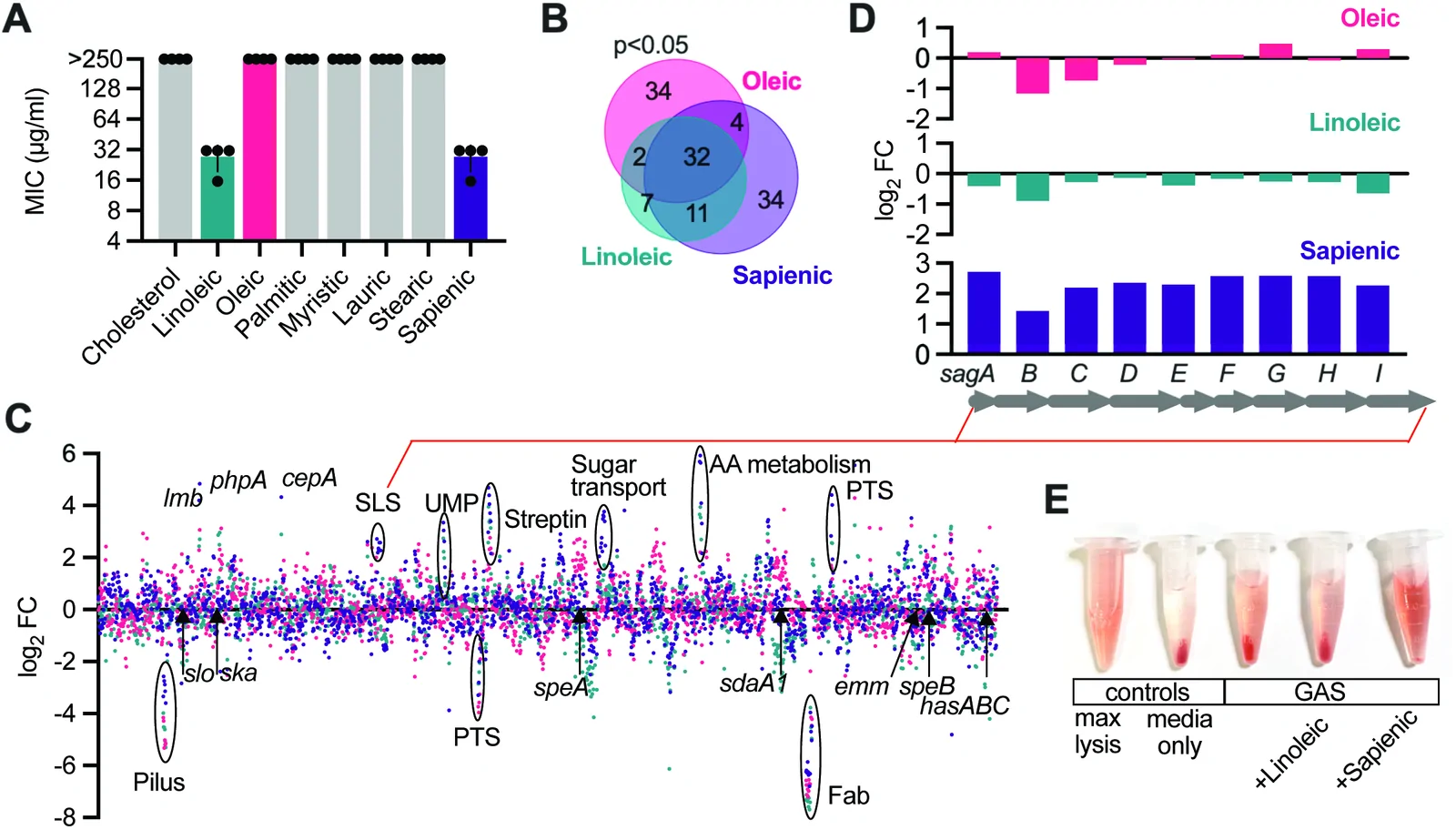
A human skin lipid, sapienic acid, induces SLS-mediated hemolysis. (**A**) GAS 5448 was incubated with serial dilutions of the major lipid species of the human skin. Experiments were performed four times; error bars represent standard deviations. (**B**) RNA-seq was performed on GAS grown with oleic, linoleic, or sapienic acids. The Venn diagram represents the number of significant hits with FDR *P*-values < 0.01 relative to the untreated control. (**C**) Log_2_FC values were calculated by comparing each treatment to the control and plotted across the genome; blue, linoleic; purple, oleic; and green, sapienic. Major upregulated and downregulated genes under lipid treatment conditions, as well as keystone virulence factors, are indicated. (**D**) Examination of the *sag* operon and its differential regulation under lipid treatment conditions relative to untreated control. (**E**) Bacterial cultures grown with linoleic or sapienic acid were incubated with purified human RBCs; hemolysis is indicated by the loss of the RBC pellet.

Therefore, we used RNA-seq to examine the impact of sub-antimicrobial concentrations of sapienic acid on GAS gene expression relative to the similarly antimicrobial FA, linoleic acid, and a non-antimicrobial FA control, oleic acid (Fig. 1B). All three FAs altered GAS gene expression (Tables S1 to S4), inducing substantially overlapping changes in expression relative to untreated bacteria (Fig. 1B), with nearly all the top differentially regulated genes encoding factors involved in fatty acid biosynthesis, consistent with bacterial scavenging of these exogenous fatty acids (Fig. 1C and Tables S1 to S4). To understand the basis for the increased inflammation seen in sapienic acid-treated mice, we focused on genes uniquely regulated by that treatment. No significant changes were observed in genes encoding proinflammatory toxins or major virulence factors of GAS, including M protein, superantigens, SpeB, capsule, or the pore-forming toxin, streptolysin O (SLO) (Fig. 1C and Tables S1 to S4). However, half of the most GAS-upregulated genes were within the *sag* locus (*sagA to sagI*) (Fig. 1D). This operon was shown by Nizet et al. to encode streptolysin S (SLS) (13), a 2.7 kDa ribosomally synthesized peptide encoded by *sagA* that is post-translationally modified by heterocycle formation mediated by the other products of the *sag* operon (14, 15). The resulting toxin is oxygen-stable and enables GAS to lyse red blood cells (RBCs) during growth on blood agar (β-hemolysis), can lyse numerous additional cell types (16–18), and contributes to tissue inflammation (13, 14, 19, 20).

We reasoned that this *sag* induction could promote hemolysis and pathogenesis. Indeed, GAS grown in sub-antimicrobial concentrations of sapienic acid produced notable hemolysis relative to controls (Fig. 1E). Quantification of heme released by RBCs showed that sapienic acid, but not linoleic acid, significantly increased hemolysis by WT 5448 GAS (Fig. S2A), without affecting growth (Fig. S2B). *sagA* mutant GAS or either FA alone failed to induce hemolysis (Fig. S2A). To determine whether sapienic acid-induced SLS contributes to increased inflammation, we epicutaneously infected mice with WT GAS or the *sagA* mutant, with or without sapienic acid. Bacterial burden in the skin was not significantly different between WT GAS- or *sagA* mutant-infected mice (Fig. 2A), but, in an SLS-dependent manner, sapienic acid increased barrier disruption measured by transepidermal water loss (Fig. 2B) and neutrophil influx seen by histology (Fig. 2C). Together, these data support the finding that sapienic acid increases SLS-induced pathogenesis and pathology.

**Fig 2:**
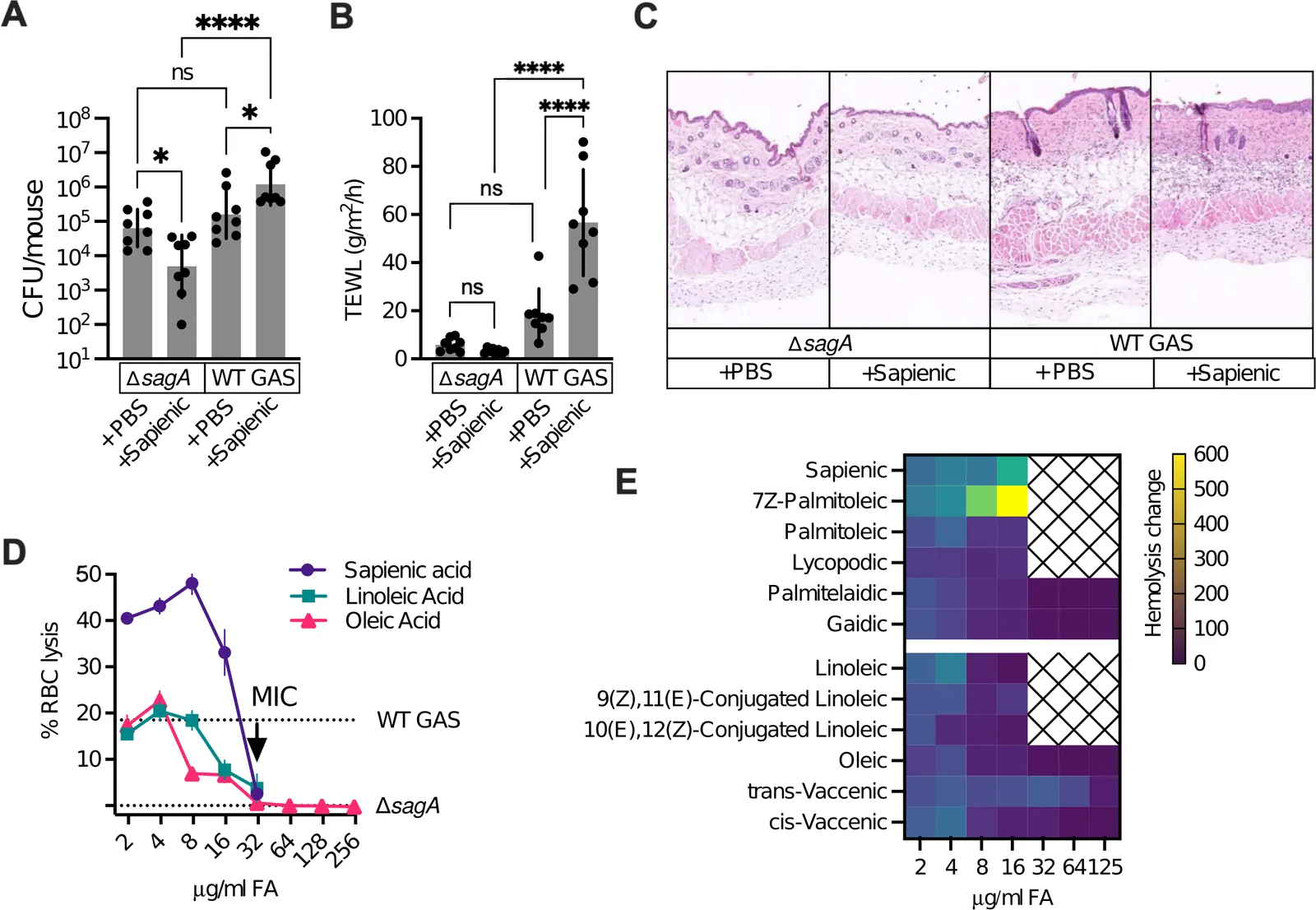
Sapienic acid worsens infection severity in mice. Mice were infected epicutaneously with GAS 5448 or Δ*sagA* (10^8^) and treated with either sapienic acid (50 μL of 1 mM) or PBS control. After 48 h, bacterial burden (**A**) and transepidermal water loss (TEWL) (**B**) were measured, and the skin (**C**) was histologically examined by hematoxylin and eosin (H&E) stain. *P < 0.05, ****P < 0.00005 (ANOVA; Tukey *post hoc* test). Experiments were performed three times; error bars represent standard deviations between the pooled animals (*n* = 8). (**D**) GAS 5448 grown in titrations of each indicated FA incubated with human RBCs, then hemolysis relative to the positive control was measured by hemoglobin release. Dashed lines represent average % RBC lysis for WT GAS or Δ*sagA* incubated without FA. (**E**) GAS 5448 grown in titrations of each indicated FA incubated with human RBCs, and hemolysis was normalized to that caused by GAS 5448 grown without any supplemented FA in parallel.

In contrast to sapienic acid, we noted that increasing concentrations of linoleic acid and oleic acid reduced hemolysis by GAS (Fig. 2D). Therefore, we examined the FA determinants contributing to antimicrobial activity and SLS induction or inhibition with a variety of FAs with similar chain lengths (C16 or C18) and saturation. 7Z-palmitoleic, palmitoleic, and lycopodic acids (all C16:1) had antimicrobial activity similar to sapienic acid, and polyunsaturated (C18:2) FAs 9(Z),11(E)-conjugated linoleic and 10(E),12(Z)-conjugated linoleic acids showed antimicrobial activity similar to linoleic acid (Fig. S2C). To evaluate whether these FAs could be antimicrobial without inducing SLS, GAS was incubated with dilutions of these FAs, and the resulting hemolytic activity was measured. Though not relevant to human infection, 7Z-palmitoleic acid (also known as 7Z-hexadecenoic and cis-7-hexadecenoic acid) induced even greater hemolysis than sapienic acid (Fig. 2E). Palmitoleic, lycopodic, linoleic, 9(Z),11(E)-conjugated linoleic, and 10(E),12(Z)-conjugated linoleic acids all reduced hemolysis, even at sub-antimicrobial concentrations (Fig. 2E). Furthermore, FAs lacking antimicrobial activity could still protect RBCs from GAS-induced hemolysis in a dose-dependent manner (Fig. 2E). Together, these data suggest that interference with SLS-induced lysis could be an impactful anti-virulence contribution of FAs during infection.

We have demonstrated that sapienic acid exhibits antimicrobial activity toward GAS *in vitro* but exacerbates disease severity *in vivo* by driving SLS production. Sapienic acid accumulates in human skin due to high conversion of palmitic acid resulting from the overexpression of fatty acid desaturase 2 (FADS2) relative to other organisms (21), and corresponding underexpression of *sag* in model organisms could lead to underestimation of its contribution to disease. Sapienic acid is reduced in atopic dermatitis lesional skin, and in humans and mice, application of sapienic acid is thought to have protective benefits that including being noninflammatory and antimicrobial against *Staphylococcus aureus* (22, 23). Unlike our results with GAS, sapienic acid did not induce *S. aureus* toxins (6). Lastly, our results highlight that linoleic acid has dual antimicrobial and antitoxin activity. Antibiotic failure is an ongoing issue in the treatment of infection, and for severe disease, treatments that interfere with toxin activity have therapeutic benefits (24). FAs are already commonly incorporated into skincare products intended for cleaning, exfoliation, reducing inflammation, and reinforcement of damaged skin. Our results suggest potential for use in infectious diseases.

## SUPPLEMENTAL MATERIAL

Supplemental Material mbio.01856-26-s0001.docx Text S1; Figures S1 and S2.

Legends mbio.01856-26-s0002.docx Legends for supplemental tables.

Table S1 mbio.01856-26-s0003.txt RNAseq, control.

Table S2 mbio.01856-26-s0004.txt RNAseq, oleic.

Table S3 mbio.01856-26-s0005.txt RNAseq, linoleic.

Table S4 mbio.01856-26-s0006.txt RNAseq, sapienic.

ASM does not own the copyrights to Supplemental Material that may be linked to, or accessed through, an article. The authors have granted ASM a non-exclusive, world-wide license to publish the Supplemental Material files. Please contact the corresponding author directly for reuse.
