# Supplementary material for: A uniquely human sebum lipid drives streptococcal skin infection severity
Source: mBio. 2026 Sep 3;17(10):e01856-26. doi: 10.1128/mbio.01856-26 (PMC13712856; doi:10.1128/mbio.01856-26)
Supplement: Supplementary file 1 [file mbio.01856-26-s0001.docx]

Supplemental material

***A uniquely human sebum lipid drives streptococcal skin infection severity***

Doris. L. LaRock^a#^, Christopher N. LaRock^ab#^

^a^Department of Microbiology and Immunology, Emory University School of Medicine, Atlanta, GA, United States of America

^b^Department of Medicine Division of Infectious Disease, Emory University School of Medicine, Atlanta, GA, United States of America

#Address correspondence to: Doris L. LaRock, doris.larock@emory.edu or

Christopher N. LaRock, christopher.larock@emory.edu

**Contents**

**Text S1.** Materials and Methods

**Figure S1.** Sapienic acid worsens infection severity in mice.

**Figure S2.** Sapienic acid induces SLS-mediated hemolysis.

**Text S1. MATERIALS AND METHODS**

***Ethics Statement.*** This study was conducted according to principles expressed in the Declaration of Helsinki. Blood was collected from healthy adult volunteers under informed consent and approved by the Institutional Review Board of Emory University.

***Bacterial strains***. GAS 5448, an M1T1 serotype strain originally isolated from a patient with necrotizing fasciitis, and its isogenic SLS mutant (Δ*sagA*) have been previously described (1, 2). All strains were grown in static liquid cultures of Todd-Hewitt Yeast (THY; Difco) or on 5% Blood Agar (Hardy Diagnostics) at 37°C and 5% CO_2_. Bacteria from overnight cultures were first washed and diluted in PBS in preparation for infection experiments.

***Lipid methods*.** Fatty acids were curated using the Lipidomics Gateway of the LIPID Metabolites And Pathways Strategy (LIPID MAPS) database. All lipids were purchased from Cayman Chemical, dissolved in chloroform to 25 mg/ml when undiluted, then dried. Before use, dried lipids were resuspended in PBS with sonication to 5 mg/ml stock and diluted as indicated for each experiment.

***Minimum inhibitory concentration***. Minimum inhibitory concentration (MIC) assays were carried out in liquid media in 96-well plates, essentially as previously (3). Briefly, GAS cultures were prepared in RPMI with phenol red + 5% THY, to which dilutions of each test lipid were added; RPMI was used throughout as a diluent in place of cation-adjusted Mueller–Hinton broth or other complex media because they can mask effects from charged or hydrophobic antimicrobials (4). The MIC was determined to be the lowest concentration that inhibited the growth of GAS after 18 hr incubation at 37°C in 5% CO_2_. A change of media color from red to yellow with corresponding turbidity was interpreted as bacterial growth, as previously (5). GAS incubated with sub-inhibitory concentrations of sapienic or linoleic acid (15.6 μg/ml) were plated on THY agar for CFU determination.

***RNAseq***. Overnight GAS cultures in RPMI with phenol red + 5% THY with 15.6 μg/ml sapienic or linoleic acid were pelleted and shipped to Genewiz. RNA prepared by Genewiz for Illumina RNA sequencing with rRNA depletion performed on each sample. Sequence reads were trimmed to remove possible adapter sequences and nucleotides with poor quality using Trimmomatic v.0.36. The trimmed reads were then mapped to the Streptococcus_pyogenes_GCF_900619585.1 reference genome available on ENSEMBL (NCBI Reference Sequence: NZ_LR130236.1) using the Bowtie2 aligner v.2.2.6. Unique gene hit counts were calculated by using featureCounts from the Subread package v.1.5.2. The hit counts were summarized and reported using the gene_id feature in the annotation file. Only unique reads that fell within gene regions were counted. Using DESeq2, a comparison of gene expression between sample groups was performed and the Wald test used to generate p-values and log2 fold changes. Genes with an adjusted p-value < 0.05 and absolute log2 fold change > 1 were called as differentially expressed genes for each comparison. Overlaps in expression patterns were visualized using the BioVenn package v.1.1.3.

***Hemolysis assay*.** SLS activity was initially observed as zones of beta-hemolysis surrounding colonies grown on 5% blood agar. Quantification of hemolytic activity was performed as previously described (6). A 2% red blood cell suspension was prepared in DPBS. Briefly, 1 mL of heparinized whole blood was washed with 20 mL DPBS and centrifuged at 1,000 x g for 10 minutes at room temperature. The wash was completed twice more. RBCs were resuspended in 50 mL DPBS and stored at 4°C until use. GAS (15 μL) from the MIC assay described previously was mixed with 100 μL RBC suspension and 85 μL PBS and incubated for 1 hour at 37°C in 96-well round bottom plates. For images, volumes were increased 5-fold and incubated in 1.7 mL Eppendorf tubes. Media alone was used as control for spontaneous lysis (A_0_), and media with 1% Triton X-100 used to determine maximum lysis (A_100_). Following incubation, the mixture was centrifuged at 1,000 x g for 10 minutes, 100 μL of supernatant was transferred to a new 96-well plate, and amount of released hemoglobin measured as absorbance at 405 nm (An) ((Victor Nivo, Perkin Elmer plate reader). Percentage of RBC lysis is defined as ((An – A_0_)/(A_100_ − A_0_)) × 100.

***In vivo infection models.*** All animal experiments were performed with prior approval from Emory University’s Institutional Animal Care and Use Committee. 8-12-week-old wild-type C57BL/6 mice were ordered from Jackson Laboratories as needed. Mice were housed under pathogen-free conditions. The day before infection, fur was removed from the back using an electric razor and hair removal cream. Pre-infection, mice were prepared by tape-striping then wiped clean with an alcohol pad, then 10^8^ CFU bacteria in 50 μL PBS were pipetted directly onto the skin as previously (7). Lipid (1 mM) or PBS carrier control were added in a 50 μL volume to a 1 cm^2^ gauze patch applied overtop, then covered with Tegaderm. Mice were monitored then euthanized after 48 h. Trans epidermal water loss (TEWL) of infected skin was measured using a Tewameter Hex (Courage + Khazaka). Post-euthanasia, skin lesions were excised and homogenized, and dilutions plated for CFU on 5% Blood Agar as previously (8), or fixed in 4% formalin, paraffin-embedded lungs, stained hematoxylin and eosin, imaged on a Nanozoomer 2.0Ht Slide Scanner (Hamamatsu), analyzed using NDP.view 2 (Hamamatsu), all as previously (9).

***Statistics*.** Statistical analyses were performed using Prism 10 (GraphPad) using 1-way or 2-way t-test or ANOVA as appropriate. No blinding or exclusion criteria were applied. Tukey post-tests were used to correct for multiple comparisons. Statistical significance is indicated as (*, P < 0.05; **, P < 0.005; ***, P < 0.0005; ****, P < 0.00005). All error bars show the mean and the standard deviation (s.d.). Data are representative of at least three independent experiments.

***Data availability.*** Raw Illumina reads from RNA-Sequencing are available under NCBI accession number GSE324382 (https://www.ncbi.nlm.nih.gov/geo/query/acc.cgi?acc=GSE324382). Reviewer token: mbkxmgiitdqxtqx

**Figure S1.** Sapienic acid worsens infection severity in mice. Mice were infected epicutaneously with GAS 5448 then linoleic or sapienic acids, or PBS control, applied. After 48 h, bacterial burden was measured by dilution plating (**A**), and tissue imaged (**B**). **P<0. 05* (ANOVA; Tukey post-test).

**Figure S2.** Sapienic acid induces SLS-mediated hemolysis. Quantification of RBC lysis by measuring released hemoglobin (**A**). Bacterial CFU quantification of GAS grown with subinhibitory sapienic, linoleic, or negative control (**B**). GAS 5448 incubated with serial dilutions of the indicated FA, and growth evaluated after 18 h incubation to determine the minimum inhibitory concentration (MIC) (**C**).

*****P<0.00005* (ANOVA; Tukey post-test). Experiments were performed four times; error bars represent standard deviations.

**REFERENCES**

1.	Datta V, Myskowski SM, Kwinn LA, Chiem DN, Varki N, Kansal RG, Kotb M, Nizet V. 2005. Mutational analysis of the group A streptococcal operon encoding streptolysin S and its virulence role in invasive infection. Molecular Microbiology 56:681–695.

2.	Kansal RG, McGeer A, Low DE, Norrby-Teglund A, Kotb M. 2000. Inverse relation between disease severity and expression of the streptococcal cysteine protease, SpeB, among clonal M1T1 isolates recovered from invasive group A Streptococcal Infection Cases. Infection and immunity https://doi.org/10/drpfcs.

3.	Wilde S, Dash A, Johnson A, Mackey K, Okumura CYM, LaRock CN. 2023. Detoxification of reactive oxygen species by the hyaluronic acid capsule of group A Streptococcus. Infection and Immunity 0:e00258-23.

4.	Lin L, Nonejuie P, Munguia J, Hollands A, Olson J, Dam Q, Kumaraswamy M, Rivera H, Corriden R, Rohde M, Hensler ME, Burkart MD, Pogliano J, Sakoulas G, Nizet V. 2015. Azithromycin Synergizes with Cationic Antimicrobial Peptides to Exert Bactericidal and Therapeutic Activity Against Highly Multidrug-Resistant Gram-Negative Bacterial Pathogens. EBioMedicine 2:690–698.

5.	Dohrmann S, LaRock CN, Anderson EL, Cole JN, Ryali B, Stewart C, Nonejuie P, Pogliano J, Corriden R, Ghosh P, Nizet V. 2017. Group A Streptococcal M1 Protein Provides Resistance against the Antimicrobial Activity of Histones. Scientific reports 7:43039.

6.	Wierzbicki IH, Campeau A, Dehaini D, Holay M, Wei X, Greene T, Ying M, Sands JS, Lamsa A, Zuniga E, Pogliano K, Fang RH, LaRock CN, Zhang L, Gonzalez DJ. 2019. Group A Streptococcal S Protein Utilizes Red Blood Cells as Immune Camouflage and Is a Critical Determinant for Immune Evasion. Cell Reports 29:2979-2989.e15.

7.	LaRock DL, Sherman JD, Qu C, Ngai W, Read TD, McGavin MJ, LaRock CN. 2025. Staphylococcus aureus induces Gasdermin A-dependent keratinocyte pyroptosis. Nat Commun 16:10570.

8.	LaRock CN, Todd J, LaRock DL, Olson J, O’Donoghue AJ, Robertson AAB, Cooper MA, Hoffman HM, Nizet V. 2016. IL-1β is an innate immune sensor of microbial proteolysis. Science immunology 1:eaah3539.

9.	Carr MA, Chou SR, Sun J, LaRock DL, LaRock CN. 2025. Therapeutic Inhibition of Metalloproteases by Tetracyclines During Infection by Multidrug-Resistant Pseudomonas. J Infect Dis 232:e416–e420.
