# Supplementary material for: A uniquely human sebum lipid drives streptococcal skin infection severity
Source: mBio. 2026 Sep 3;17(10):e01856-26. doi: 10.1128/mbio.01856-26 (PMC13712856; doi:10.1128/mbio.01856-26)
Supplement: Supplementary file 2 [file mbio.01856-26-s0002.docx]

Supplemental material

***A uniquely human sebum lipid drives streptococcal skin infection severity***

Doris. L. LaRock^a#^, Christopher N. LaRock^ab#^

^a^Department of Microbiology and Immunology, Emory University School of Medicine, Atlanta, GA, United States of America

^b^Department of Medicine Division of Infectious Disease, Emory University School of Medicine, Atlanta, GA, United States of America

#Address correspondence to: Doris L. LaRock, doris.larock@emory.edu or

Christopher N. LaRock, christopher.larock@emory.edu

**LEGENDS FOR SUPPLEMENTAL TABLES**

**Table 1.** RNAseq of untreated control GAS 5448

**Table 2.** RNAseq of oleic acid-treated GAS 5448

**Table 3.** RNAseq of linoleic acid-treated GAS 5448

**Table 4.** RNAseq of sapienic acid-treated GAS 5448
